# Visits to the Pediatric Emergency Department for Eye Conditions Before and During the COVID-19 Pandemic

**DOI:** 10.5811/westjem.2022.1.53392

**Published:** 2022-03-24

**Authors:** Jing Jin, Lauren Bules, Kaynan Doctor, Dorothy Hendricks, Katherine Callaghan, Julia E. Reid, Jonathan Salvin, Sharon Lehman, Airaj Fasiuddin, Joseph Piatt

**Affiliations:** * Nemours Children’s Hospital, Division of Ophthalmology, Wilmington, Delaware; † Lewis Katz School of Medicine at Temple University, Philadelphia, Pennsylvania; ‡ Nemours Children’s Hospital, Division of Emergency Services, Wilmington, Delaware; § Nemours Children’s Hospital, Department of Pediatrics, Wilmington, Delaware; ¶ Children’s Hospital of Philadelphia, Division of Ophthalmology, Philadelphia, Pennsylvania; || Nemours Children’s Hospital, Division of Ophthalmology, Orlando, Florida; # Nemours Children’s Hospital, Division of Neurosurgery, Wilmington, Delaware

## Abstract

**Introduction:**

The use of the emergency department (ED) has been increasing, and many visits occur for non-urgent conditions. A similar trend was found among adult visits to the ED for ocular conditions. In this study we analyzed the impact of sociodemographic factors, presentation timing, and the COVID-19 pandemic on pediatric ED (PED) encounters for ophthalmologic conditions. It is important to identify the multifold factors associated with overutilization of the ED for non-urgent conditions. Caring for these patients in an outpatient clinical setting is safe and effective and could decrease ED crowding; it would also prevent delays in the care of other patients with more urgent medical problems and lower healthcare costs.

**Methods:**

We retrospectively reviewed electronic health records of PED ocular-related encounters at two children’s hospitals before (January 2014–May 2018) and during the COVID-19 pandemic (March 2020–February 2021). Encounters were categorized based on the International Classification of Diseases codes into “emergent,” “urgent,” and non-urgent” groups. We analyzed associations between sociodemographic factors and degrees of visit urgency. We also compared visit frequencies, degrees of urgency, and diagnoses between pre-pandemic and pandemic data.

**Results:**

Pre-pandemic ocular-related PED encounters averaged 1,738 per year. There were highly significant sociodemographic associations with degrees of urgency in PED utilization. During the 12-month pandemic timeframe, encounter frequency contracted to 183. Emergent visits decreased from 21% to 11%, while the proportions of urgent and non-urgent encounters were mostly unchanged. The most common pre-pandemic urgent diagnosis was corneal abrasion (50%), while visual disturbance was most common during the pandemic (92%). During both time periods, eye trauma was the most frequent emergent encounter and conjunctivitis was the most common non-urgent encounter.

**Conclusion:**

Sociodemographic factors may be associated with different types of PED utilization for ocular conditions. Unnecessary visits constitute major inefficiency from a healthcare-systems standpoint. The marked decrease in PED utilization and differing proportions of ocular conditions encountered during the pandemic may reflect a decrease in incidence of many of those conditions by social distancing; these changes may also reflect altered parental decisions about seeking care.

## INTRODUCTION

Emergency departments (ED) provide acute and after-hours care to millions of Americans each year. Patients’ use of EDs has risen rapidly from 108 million visits in 2000 to 130 million in 2010.^1^ In 2015, over 16% of children in the United States visited an ED at least once.^2^,^3^ A 2013 systematic review by Uscher-Pines et al reported that nearly 40% of all ED visits occurred for non-urgent medical conditions.^4^ Studies demonstrate several characteristics associated with this type of ED use including younger age, Black race, and lower patient income.^4^

Channa et al reported a similar trend within the field of ophthalmology. Using the US Nationwide Emergency Department Sample, they found that over 40% of ED visits for ocular conditions were non-urgent.^5^ Additionally, Stagg et al investigated factors affecting adult patient visits to several EDs for ocular conditions,^6^ concluding that nearly one-quarter presented for non-urgent ocular problems. These encounters shared similarities with non-ophthalmological, non-urgent medical conditions, as they were more commonly associated with younger age groups, Black and Hispanic populations, lower income, and male gender.

There is no robust literature describing the characteristics of pediatric patients receiving emergency eye care, based on electronic health records (EHR) data for ocular conditions. When our study was nearing completion, an unanticipated event presented a unique opportunity. First identified in December 2019, coronavirus disease 2019 (COVID-19) rapidly spread across the globe. Implementation of stay-at-home orders along with school and workplace closures significantly altered families’ daily routines. Given this situation, we extended our study to examine whether the COVID-19 pandemic affected the etiologies of ocular conditions and perceptions of urgency leading to PED visits.

## METHODS

### Data Source

The EHR dataset of our institution contains information on ED encounters including ≥ 1 *International Classification of Diseases*, 9^th^ and 10^th^ revisions, *Clinical Modification* (ICD-9-CM and ICD-10-CM) as the primary diagnosis for ED visits. We included in the pre-pandemic dataset all patients < 21 years who presented to our PEDs in Delaware and Florida between January 1, 2014–May 31, 2018, while March 1, 2020–February 28, 2021 constituted the pandemic dataset. We searched records using ICD-9-CM and ICD-10-CM codes denoting diseases of the eye and adnexa, and eye trauma. For each patient encounter, we had access to medical records for ocular and non-ocular conditions as well as sociodemographic information including age, gender, race, ethnicity, and insurance type. We stratified patient age groups according to parameters described in recent literature^7^ with adjustment as follows: neonates (less than one month); infants (one month up to one year); preschool (one year up to five years); school age (5–13 years); and teen (13 years or greater).

**Population Health Research Capsule**

What do we already know about this issue?

*Emergency department (ED) visits have been increasing, and many are for non-urgent conditions. A similar trend was found among adults visiting EDs for eye diseases.*

What was the research question?

*What are the factors related to pediatric ED visits for non-urgent ophthalmic conditions before and during the COVID-19 pandemic?*

What was the major finding of the study?

*ED visits for eye complaints declined 89.5% during the pandemic year period.*

How does this improve population health?

*Caring for non-urgent conditions at outpatient clinics is safe and effective. It may prevent ED crowding and delayed care for urgent conditions, and decrease healthcare costs.*

The institutional review board of our institution approved this study.

### Reasons for Visits to the Emergency Department for Ocular Conditions

Recently, Stagg and colleagues classified ocular conditions with ICD-9-CM codes into three categories: “non-urgent;” “urgent;” or “other.”^6^ Prior to data analysis, our panel of pediatric ophthalmologists modified these criteria and defined ophthalmological diagnoses as “non-urgent,” “urgent,” or “emergent.” “Non-urgent” conditions were unlikely to affect visual acuity or cause considerable discomfort necessitating urgent medical attention. Patients with non-urgent conditions could seek care safely in outpatient office settings. “Emergent” ocular conditions (ie, eye trauma) were sight- or life-threatening and associated with decreased visual acuity, caused severe pain, or constituted an indication for immediate surgical intervention. This definition was synonymous with the “urgent” classification found in the Stagg study. We classified the remaining ocular conditions as “urgent,” a group similar to Stagg’s “other” category. An example diagnosis within this group is corneal abrasion, which, despite quick healing without sequelae, causes intense pain and occasional blurred vision that may reasonably justify a visit to the ED.

Table 1 lists the most frequent ocular diagnoses and associated ICD-9-CM and ICD-10-CM codes captured in this study. When an encounter had multiple diagnoses, it was classified by that of highest acuity. Finally, for those with multiple visits we classified each visit for that patient individually based on diagnoses. We also explored other variables including encounter date and time to assess whether accessibility to outpatient care and seasonal variations affected our data.

### Data Analyses

For the pre-pandemic data, we analyzed univariate associations of demographic factors with the urgency of the PED visits by cross-tabulation and the Pearson chi square test. Multivariate associations were assessed by multinomial logistic regression. We developed two models: 1) comparing urgent visits with non-urgent visits; and 2) comparing emergent visits with non-urgent visits. The following covariates were included initially in both models: age group; gender; payer; race/ethnicity; state; and all their second-order interactions. Final models were constructed after excluding covariates and interactions that did not retain significance at the *P* < 0.05 level. We calculated adjusted odds ratios (aOR) and 95% confidence intervals (CI). Hypothesis testing was performed using a Type III Wald chi-squared statistic. Model fit was analyzed with the Hosmer-Lemeshow test, and model discrimination was determined with the C-statistic. We performed a complete-case analysis. Only first visits for each patient were analyzed to maintain independence of observations.

Only descriptive data are presented for the pandemic dataset due to the small number of observations. Data were securely compiled in Excel (Microsoft Corporation, Redmond, WA) and analyzed using RStudio (R Foundation for Statistical Computing, Vienna, Austria).

## RESULTS

In the pre-pandemic epoch, 7077 patients visited our PEDs 7675 times with ophthalmologic complaints, comprising 1.9% of total PED visits (Figure A). During this period, the total number of visits to the PED increased year by year; however, visits due to ocular conditions remained constant at approximately 2%. Throughout the pandemic period, the total number of PED visits decreased to 60% of the pre-pandemic average annual PED visit rate (Figure B). Visits due to ocular conditions decreased to 0.34% (*P* = 0.002).

### Pre-Pandemic Characteristics of Enrollees Seeking Care in a Peds ED for Ocular Conditions

Sample characteristics and their univariate associations with visit urgency are presented in Table 2. All factors were highly associated with certain levels of urgency except office hours. Urgency increased with patient age. White children had proportionately more urgent visits. Males had more urgent visits than females. Commercially insured children were far more likely to visit for urgent and emergent indications compared with children who had public insurance or no insurance. Visits to the Delaware ED were generally more urgent than visits in Florida.

To learn whether our study population differed from the residents of the communities surrounding our hospitals, we compared our dataset with local population race/ethnicity information in the US Census Bureau database. Our patients in the non-urgent visit category had a lower proportion of White patients (35.6%) than the proportion of White inhabitants seen within the catchment areas (49.8%). The proportions of patients who self-identified as Black (32.8%) and Hispanic (24.2%) were greater than those of the local populations (26.1% and 20.2%, respectively).

### Multivariate Analysis of Pre-Pandemic Encounter Urgency

Multivariate associations of demographic factors with urgent and emergent visits are presented in Table 3. Table 3a compares urgent encounters with non-urgent encounters. Table 3b compares emergent encounters with encounters of lesser urgency. All factors significantly associated with encounter urgency in the univariate analysis retained significance in the multivariate models. The urgency of ED encounters increased in a monotonic fashion with patient age. Urgent encounters were most frequent for commercially insured patients. White patients from Delaware were much more likely to visit for urgent and emergent reasons than patients of other races/ethnicities in Delaware and all patients in Florida. Black patients in Florida had more frequent urgent and emergent visits than Black patients in Delaware. The opposite was true for Hispanic patients. Patient gender did not retain a significant association with visit urgency in multivariate analysis.

### Pre-Pandemic Repeat Peds Emergency Department Visits for Ocular Conditions

Of the 7,077 patients who visited the PED for ocular problems in the pre-pandemic period, 500 (7.1%) visited the PED more than once for ophthalmologic conditions (Table 4). First and second visits for patients who frequented the ED often shared the same ophthalmological diagnosis if the duration between visits was less than 14 days. As a group, rapid second return visits were associated with lower acuity compared with first visits. Second visits occurring after a longer interval (>14 days) were more likely to be associated with higher acuity/urgency compared with corresponding first visits.

### Comparison of Ocular Conditions in PED Encounters Before and During the Pandemic

Rates of PED encounters for ocular conditions during the pandemic were far lower than pre-pandemic rates, but not all conditions were equally affected. In Table 5, average annual pre-pandemic rates are compared with actual encounters during the pandemic year for each of the more common conditions. The differences between pre-pandemic averages and actual pandemic encounters are tabulated as “missing encounters.” Emergent ocular conditions were all comparably decreased. Urgent encounters were almost eliminated except for encounters for visual disturbances, which appeared unaffected. Observations regarding non-urgent conditions were mixed: encounters for conjunctivitis were greatly diminished and disappeared entirely for eye area edema. Encounters for hordeolum were not affected.

## DISCUSSION

The COVID-19 pandemic presented an unprecedented circumstance affecting all Americans, altering the types and frequencies of ocular conditions presenting to our PEDs. Marked reductions in overall PED encounters were observed once the declaration of national emergency occurred. Our detailed characterization of pre-pandemic ocular-related PED visits serves as a basis for discussion on the effects of the pandemic. In the pre-pandemic epoch, there were over 7000 encounters within our PEDs for ocular problems, which increased in both locations year by year. These represented 2% of all PED visits throughout the study period. Approximately 60% of these encounters were classified for non-urgent ophthalmological conditions.

### Sociodemographic Factors and Urgency of Pediatric Emergency Department Encounters

In multivariate analysis, demographic factors including younger age, Black race, Hispanic ethnicity, and use of public health insurance or no insurance were positively associated with PED encounters for non-urgent ocular conditions. These findings are consistent with the adult ED literature.^6^ The association of age with encounter urgency likely reflects several factors. High parental anxiety may account for the low average urgency of PED encounters in the neonatal and infant age groups. McDermott et al reported that increasing prevalence of traumatic conditions among older children may contribute to the trend toward higher urgency among older patients; our observations corroborate this conclusion.^3^ In discordance with expectations based on existing literature, male gender was not associated with visit urgency in multivariate analysis. Previous studies revealed 65% of all eye injuries and 75% of sports and recreation-related eye injuries presenting to the ED were seen among male pediatric and adolescent patients.^9^

Our observations of the effects of race, ethnicity, and healthcare payer are consistent with many previous reports of ED resource utilization. Stagg et al demonstrated that less affluent patients and ethnic minorities are more likely to present to the ED for non-urgent ocular problems regardless of insurance status.^6^ Outside the field of ophthalmology, McDermott et al reported that Medicaid is the expected primary payer for more than 60% of PED encounters, and other authors note the association of low income and Black race with ED utilization.^3^,^10^,^11^ Explanations for these associations are likely multifactorial. Patients of lower socioeconomic status may lack access to outpatient ophthalmology clinics. A lack of understanding about which symptoms require urgent attention may also play a role. Parental assessment of medical conditions can be especially difficult with young children, but the current analysis did not demonstrate any interaction between healthcare payer and age group. Other potential reasons for use of the PED over outpatient options may include absence of an established pediatric medical home and challenges with public transportation.

### Time and Urgency of Pediatric Emergency Department Encounters

We hypothesized that low-urgency encounters would be more frequent outside regular clinic office hours. Work responsibilities of parents and families may make scheduling appointments during ophthalmology clinic hours impossible. During the pre-pandemic epoch, weekday clinic hours were not associated with overall frequency of encounters for ocular conditions. However, during holidays and weekends, a significantly higher proportion of PED encounters for non-urgent ocular conditions occurred.

### Repeat Encounters

In the pre-pandemic epoch, approximately 7.1% of patients in our study visited more than once for ocular conditions. Over 50% of second return encounters occurred within five days of the first. Conjunctivitis was the most frequent cause for all PED encounters and constituted the reason for more than 70% of non-urgent encounters. The natural course of infective conjunctivitis is approximately 14 days;^12^ parental impatience with the pace of recovery could account for repeat visits. Patient and provider education may reduce rates of return encounters.

### Interpretation of Pandemic Encounter Data

The COVID-19 pandemic altered the patterns of family life in profound ways, and it may have affected parental decisions about the urgency of children’s medical conditions and risk for a visit to PED. Both factors may be reflected within our dataset. Traumatic ocular conditions essentially vanished from the PED during the pandemic. These changes undoubtedly reflect the suspension of athletic activities and decreased outdoor play with children from other families. Conjunctivitis almost disappeared from our PEDs as well. The closure of childcare facilities likely interrupted the transmission of this highly contagious disease. Conversely, visits for visual disturbances and hordeolum continued at low but steady rates, seemingly unaffected by the pandemic.

### Operational Implications

Most of the urgent and non-urgent conditions tabulated in this study could have been managed in the ophthalmology office with better stewardship of material and personnel resources. Stagg et al suggests incentivizing eye care physicians to offer after-hours eye care clinics as a possible solution.^6^ Utilization of teleophthalmology for triage of patients with eye conditions could reduce the number of unnecessary ED visits for ocular problems. Multidisciplinary case management efforts directed at frequent ED visitors may also reduce low-acuity encounters. Unnecessary PED visits for conjunctivitis, by far the most common diagnosis in this study, might be addressed by education to enhance the confidence of parents and primary care physicians in the management of this condition.

## LIMITATIONS

Our study, the first of its kind, was based on the EHR of a large, geographically diverse children’s healthcare system. Clinical as well as administrative data were accessible, and our observations are likely generalizable. The large sample size for the pre-pandemic epoch allowed for modeling to account for associations between encounter urgency and a variety of covariates.

The highly significant interaction between race/ethnicity and state in the pre-pandemic epoch is unexplained. It likely reflects referral patterns and local availability of alternative sources of care. The Delaware ED has a fairly distinct catchment area between Philadelphia and Baltimore. The Florida ED is relatively close to two other institutions offering pediatric emergency services. Further exploration of this interaction might reveal important disparities in access to primary and specialty care, but a population-based study of regional demographics and clinicians was far beyond the scope of the current project.

The decrement in PED encounters for ocular conditions during the pandemic precluded statistical analysis. Only qualitative comparisons with our extensive pre-pandemic dataset were possible. The data for our study was identified by diagnosis codes generated by clinicians in the ED. Most cases were not confirmed by eye care professionals, and full medical records were not reviewed in detail.

## CONCLUSION

Before and during the COVID-19 pandemic, approximately 60% of patients visiting our pediatric emergency departments for ocular complaints received non-urgent diagnoses. Our analyses indicated that younger age groups, Black patients, Hispanic patients, and families with public health insurance or no insurance were more likely to visit the PED for a non-urgent eye condition. Policymakers, insurers, healthcare administrators, and clinicians should focus future efforts on directing patients with non-urgent ocular diagnoses to other settings besides the PED.

## Figures and Tables

**Figure:**
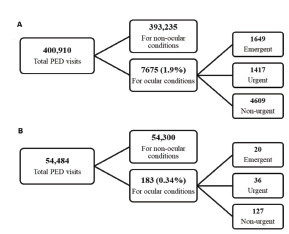
Visits to the pediatric emergency departments (PED). Number of visits January 1, 2014–May 31, 2018 (A), and March 1, 2020–February 28, 2021 (B).

**Table 1:** *International Classification of Disease*s codes used to define urgency categories of ocular diagnoses made during emergency department visits.

Ocular diagnosis	ICD-9-CM	ICD-10-CM
Emergent		
Eyebrow laceration	873.42	S01.81XA
Eye injury/trauma	871.9	S05.90XA
	918.9	
Eyelid laceration	870.0	S01.119A
	870.2	
Urgent		
Corneal abrasion	918.1	S05.00XA
Eye pain	379.91	H57.10
Visual disturbance	368.8	H53.8
Contusion of eye area	921.0	S00.10XD
	921.1	
Non-urgent		
Conjunctivitis	372.30	H10.9
	372.39	H10.89
Hordeolum	373.11	H00.01
Edema of eye area	374.82	H02.849

*ICD-9-CM*, International Classification of Diseases, 9th Revision, Clinical Modification; *ICD-10-CM*, International Classification of Diseases, 10th Revision, Clinical Modification.

**Table 2:** Sample characteristics in relation to urgency of emergency department visit.

Factor	Level	Non-urgent	Urgent	Emergent	Total	*P*-value
Age group						< 0.001
	Neonate	656 (82)	251 (12)	48 (6)	801	
	Infant	123 (91)	9 (7)	3 (2)	135	
	Preschool	2,413 (65)	485 (13)	800 (22)	3,698	
	School	1,111 (50)	570 (26)	530 (24)	2,213	
	Teen	304 (37)	251 (30)	273 (33)	828	
Race						< 0.001
	White	1,643 (50)	747 (23)	928 (28)	3,318	
	Black	1,509 (68)	329 (15)	379 (17)	2,219	
	Hispanic	1,114 (71)	229 (15)	229 (15)	1,572	
	Other/unknown	341 (60)	107 (19)	118 (21)	566	
Gender						< 0.001
	Female	2,165 (65)	612 (18)	561 (17)	3,339	
	Male	2,441 (56)	800 (18)	1,093 (25)	4,335	
Payer						< 0.001
	Commercial	1,007 (39)	672 (26)	916 (35)	2,595	
	Public/none	3,600 (71)	740 (15)	738 (15)	5,080	
Hospital						< 0.001
	Delaware	2,875 (57)	974 (19)	1,194 (24)	5,045	
	Florida	1,673 (66)	422 (17)	427 (17)	2,522	
Office						0.35
	Open	1,286 (58)	438 (20)	502(23)	2,236	
	Closed	2,886 (59)	896 (18)	1,069 (22)	4,851	

Values are counts (percentages). All factors were highly associated with visit urgency except office hours.

**Table 3a:** Multinomial logistic regression modeling the probability of “urgent” visits.

Covariate	Levels	aOR (95% CI)	*P*-value
Intercept		1.040 (0.833 – 1.299)	0.73
Age			
	Neonate	0.191 (0.144 – 0.253)	< 0.001
	Infant	0.095 (0.047 – 0.192)	< 0.001
	Preschool	0.262 (0.215 – 0.321)	< 0.001
	School	0.618 (0.505 – 0.758)	< 0.001
	Teen	reference	
Payer			
	Public/none	reference	
	Commercial	2.289 (1.989 – 2.634)	< 0.001
Race-state			
	White – DE	reference	
	Black – DE	0.454 (0.378 – 0.545)	< 0.001
	Hispanic – DE	0.451 (0.353 – 0.574)	< 0.001
	Other/unknown – DE	0.545 (0.393 – 0.756)	< 0.001
	White – FL	0.553 (0.453 – 0.674)	< 0.001
	Black – FL	0.632 (0.437 – 0.915)	0.015
	Hispanic – FL	0.379 (0.295 – 0.487)	< 0.001
	Other/unknown – FL	0.521 (0.363 – 0.748)	< 0.001

Hosmer Lemeshow χ2 = 7.9872, df = 8; *P* = 0.435; c-statistic = 0.66 (0.65–0.68).

*CI*, confidence interval; *DE*, Delaware; *FL*, Florida; *aOR*, adjusted odds ratio.

**Table 3b:** Multinomial logistic regression modeling the probability of “emergent” visits.

Covariate	Levels	aOR (95% CI)	*P*-value
Intercept		1.019 (0.818 – 1.269)	0.87
Age			
	Neonate	0.090 (0.064 – 0.128)	< 0.001
	Infant	0.031 (0.010 – 0.100)	< 0.001
	Preschool	0.409 (0.367 – 0.496)	< 0.001
	School	0.539 (0.439 – 0.662)	< 0.001
	Teen	reference	
Payer			
	Public/none	reference	
	Commercial	3.091 (2.704 – 3.534)	< 0.001
Race-state			
	White – DE	reference	
	Black – DE	0.432 (0.363 – 0.514)	< 0.001
	Hispanic – DE	0.405 (0.319 – 0.513)	< 0.001
	Other/unknown – DE	0.455 (0.332 – 0.623)	< 0.001
	White – FL	0.466 (0.385 – 0.565)	< 0.001
	Black – FL	0.524 (0.362 – 0.758)	< 0.001
	Hispanic – FL	0.319 (0.249 – 0.409)	< 0.001
	Other/unknown – FL	0.412 (0.287 – 0.591)	< 0.001

Hosmer Lemeshow χ2 = 5.1897, df = 8; *P* = 0.737; c-statistic = 0.70 (0.69–0.72).

*CI*, confidence interval; *DE*, Delaware; *FL*, Florida; *aOR*, adjusted odds ratio.

**Table 4:** Comparative urgency of first and second visits.

Factor	Category	Non-urgent (%)	Urgent (%)	Emergent (%)	Total	*P*-value
Repeat visit order						< 0.001
	First	4,172 (59)	1,334 (19)	1,571 (22)	7,077	
	Second	370 (74)	64 (13)	66 (13)	500	
Interval		Greater	Lesser	Same	Total	< 0.021
	≤14 days	7 (6)	15 (13)	96 (81)	118	
	Longer	58 (15)	56 (15)	268 (70)	382	

**Table 5:** Pediatric emergency department encounters for ocular conditions before and during the COVID-19 pandemic by urgency and condition.

Urgency	Condition	Average annual pre-pandemic encounters	Pandemic encounters	“Missing” encounters (% of expected)
Emergent				
	Eyebrow laceration	178	7	171 (96)
	Eye injury/Eye trauma	81	4	77 (94)
	Eyelid laceration	78	5	73 (94)
Urgent				
	Corneal abrasion	178	2	176 (99)
	Eye pain	39	0	39 (100)
	Visual disturbance	30	33	None
	Contusion of eye area	20	0	20 (100)
Non-urgent				
	Conjunctivitis	815	76	739 (91)
	Hordeolum	86	50	36 (42)
	Edema of eye area	47	0	47 (100)
